# A p-type multi-wall carbon nanotube/Te nanorod composite with enhanced thermoelectric performance

**DOI:** 10.1039/c7ra13572f

**Published:** 2018-02-27

**Authors:** Dabin Park, Hyun Ju, Taeseob Oh, Jooheon Kim

**Affiliations:** School of Chemical Engineering & Materials Science, Chung-Ang University Seoul 06974 Republic of Korea jooheonkim@cau.ac.kr

## Abstract

In this study, multi-walled carbon nanotube (MWCNT)/tellurium (Te) nanorod composites with various MWCNT contents are prepared and their thermoelectric properties are investigated. The composite samples are prepared by mixing Te nanorods with surface-treated MWCNTs. Te nanorods are synthesized by solution phase mixing using polyvinylpyrrolidone (PVP). The MWCNTs used in this study are surface-treated with a solution consisting of H_2_SO_4_ and HNO_3_. With increasing MWCNT content, the composite samples exhibit a reduction in the Seebeck coefficient and enhanced electrical conductivity. The maximum power factor of 5.53 μW m K^−2^ is observed at 2% MWCNT at room temperature. The thermal conductivity of the composite reduced after the introduction of MWCNTs into the Te nanorod matrix; this is attributed to the generation of heterostructured interfaces between MWCNTs and the Te nanorods. At room temperature, the composites containing 2% MWCNTs exhibited the maximum thermoelectric figure of merit (*ZT*), which is ∼3.91 times larger than that of pure Te nanorods.

## Abstract 2

In this study, multi-walled carbon nanotube (MWCNT)/tellurium (Te) nanorod composites with various MWCNT contents are prepared and their thermoelectric properties are investigated.

## Graphical abstract

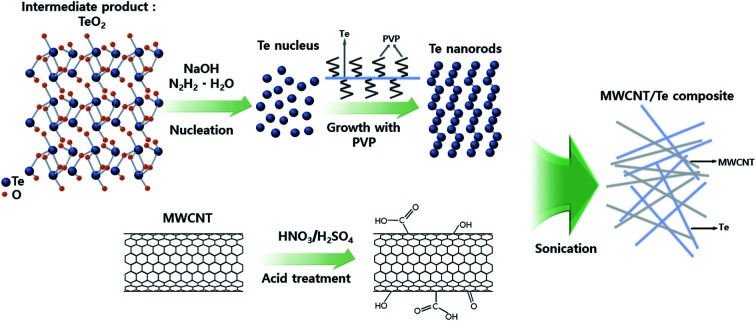

## 1. Introduction

Carbon-based fossil fuels are gradually being depleted; hence, alternative sources of energy have attracted significant interest. Some renewable energy conversion devices, such as piezoelectric, thermoelectric, fuel cells and solar cells have already been developed. Among these, thermoelectric (TE) heating and cooling devices, which are able to directly convert thermal energy into electrical energy or *vice versa*, are quite effective and exhibit potential for energy harvesting applications. The efficiency of TE materials is determined by a dimensionless number called the figure of merit (*ZT*), which can be expressed as *ZT* = *S*^2^*σT*/*κ*, where *S*, *σ*, *κ*, and *T* are the Seebeck coefficient, electrical conductivity, thermal conductivity, and absolute temperature, respectively. Theoretically, to obtain a high *ZT* value, TE materials must have a high electrical power factor (PF = *S*^2^*σ*) and low thermal conductivity.

One of the simplest ways to achieve a high PF is by using materials with high Seebeck coefficients. In general, inorganic semiconductors are promising TE materials due to their intrinsically high Seebeck coefficients owing to their crystalline structures.^1,2^ Many reports are available on inorganic semiconductors and their alloys with high *ZT* values. Nowadays, tellurium (Te) and its alloys are widely used as thermoelectric devices due to its outstanding Seebeck coefficient.^3–6^ Te is a p-type semiconductor with a narrow band gap (a direct band gap of approximately 0.35 eV at room temperature^7^) and a high Seebeck coefficient (∼400 μV K^−1^ at room temperature^8,9^). However, Te is not entirely suitable to achieve a high power factor because Te has a relatively low electrical conductivity (∼10 S m^−1^ at room temperature^10^). Furthermore, inorganic semiconductors, including Te, still face some drawbacks apart from low electrical conductivity such as mechanical/chemical instability and lack of raw materials.^11^

This limitation has driven many researchers to investigate ways of enhancing the electrical conductivity of Te.^12,13^ One of the simplest ways of enhancing the electrical conductivity of a material is to fabricate its composites with other materials. Carbon nanotubes (CNTs) offer many advantages, such as chemical stability,^14^ mechanical robustness,^15^ and high electrical conductivity.^16^ CNTs also exhibit excellent thermoelectric properties owing to their nanoscale, low dimensional, and holey structural features.^17^ Recently, many reports have been published on the enhancement of thermoelectric properties of composite materials by co-synthesis with carbon-based materials.^18–22^ Examples include the addition of single-walled carbon nanotubes (SWCNTs) to a Bi_2_Te_3_ matrix to enhance its thermoelectric properties, incorporation of graphene in a Bi_2_Te_3_ nanowire matrix to enhance its electrical conductivity while reducing the thermal conductivity,^23^ and the addition of SWCNTs to Ag_2_Te to enhance its power factor.^24^ In the case of multi-walled carbon nanotubes (MWCNTs), Khasimsaheb *et al.*^25^ reported an enhancement of power factor and *ZT* of PbTe nanocubes upon the addition of MWCNT. Similarly, Kim *et al.*^26^ synthesized MWCNT/Bi_2_Te_3_ composites and analyzed their thermoelectric properties.

A low thermal conductivity is desired for achieving a high *ZT*. Recently, phonon scattering was found to be advantageous for improving the performance of TE materials.^5,27^ Phonon scattering is increased in nanostructures, which limit the mean free path of phonons but not that of electrons; therefore, nanostructuring can reduce the lattice thermal conductivity without affecting the electrical conductivity.^5^ One-dimensional (1D) nanostructures experience increased lattice scattering of phonons, which leads to a low thermal conductivity.^28^ Therefore, the nanostructuring of materials is one way to reduce their thermal conductivity. In previous studies, it has been shown that 1D nanostructured TE materials exhibit lower thermal conductivities than their corresponding bulk structures.^5,29^ Yang *et al.*^30^ reported a reduction in the thermal conductivity of Bi_2_Te_3_ nanowires fabricated by solution-phase synthesis. Another study reported that composites of 1D nanostructures of inorganic materials and carbon-based materials exhibit high thermoelectric properties as observed in the case of graphene/Bi_2_Te_3_ composites.^31^

In this study, MWCNT/Te nanorod composite were fabricated to achieve enhanced thermoelectric properties. The Te nanorods were fabricated using a Te precursor solution *via* a polyvinylpyrrolidone (PVP)-assisted solution phase mixing process. The MWCNTs were surface-treated with an acidic solution to improve their reactivity. After acid treatment, carboxyl groups, which can improve the reactivity of the MWCNTs, were formed on the surfaces of the nanotubes. After the reaction of each materials, MWCNT/Te nanorod composites with varying MWCNT contents were synthesized. The thermoelectric properties, such as *S*, *σ*, *κ*, power factor and *ZT* of the composite samples were characterized as functions of the MWCNT content. We expect that the fabrication of Te nanorods on surface-treated MWCNTs affect each other and improve the thermoelectric properties of the composites.

## 2. Experimental

### 2.1 Materials

Hydrazine monohydrate (N_2_H_4_·H_2_O, 80%), ethylene glycol (EG, C_2_H_6_O_2_, 99.5%), sodium hydroxide (NaOH, 98%), tellurium(iv) oxide (TeO_2_, 99%), ethanol (C_2_H_5_OH, 94%), nitric acid (HNO_3_, 60%), and sulfuric acid (H_2_SO_4_, 95%) were purchased from Daejung Chemicals & Metals Co. (Seoul, Korea), PVP (molecular weight (MW) = ∼40 000) was purchased from Sigma-Aldrich (St. Louis, USA). The multi-walled carbon nanotubes used in this study were supplied by Hanwha Nanotech (Seoul, Korea).

### 2.2 Synthesis of Te nanorods

Te nanorods were prepared by mixing 1.92 g of TeO_2_ (MW = 159.6), 2.4 g of NaOH (MW = 40), and 0.8 g of PVP in a round-bottomed flask, to which 80 mL of EG was subsequently added. This solution was stirred and heated to 393 K and was followed by the injection of 4.9 mL of N_2_H_4_·H_2_O into the mixture. Due to the formation of tellurium oxide colloids, the color of the mixture turned white within a few seconds. After the injection of N_2_H_4_·H_2_O, the color of the solution turned dark gray. The reaction was allowed to continue for 90 min, after which a dark blue solution of Te nanorods was obtained. The resulting mixture was poured into deionized (DI) water containing 10 vol% hydrazine monohydrate and stirred vigorously to eliminate any residual surfactants and reactants. The resulting solution was subsequently centrifuged with the addition of volumetric water twice to remove the supernatant and re-dispersed at least twice to increase the purity of the resulting powder. Finally, the obtained product was dried in a vacuum oven at 333 K for 24 h.

### 2.3 Preparation of surface-treated MWCNTs

Pristine MWCNTs (3.0 g) were stirred in 800 mL of a mixed solution of H_2_SO_4_ and HNO_3_ (3 : 1 v/v ratio). Subsequently, this solution was heated to 353 K and stirred for 6 h. The purpose of this chemical functionalization step was to ensure that carboxyl groups were attached to the surfaces of the CNTs. The functionalized CNTs were centrifuged several times and filtered with DI water until the pH was neutral. They were then dried in a vacuum oven at 333 K for 24 h.

### 2.4 Fabrication of MWCNT/Te nanorod composites

Initially, 0.1 g of the Te nanorods were suspended in 50 mL of ethanol containing 1, 2, 3, 5, and 10 wt% of MWCNTs. Subsequently, the mixtures were subjected to ultrasonication for 30 min. Later, the mixtures were stirred at 353 K for 1 h with an appropriate amount of N_2_H_4_·H_2_O to reduce dispersion. The resulting products were washed and filtered with DI water, and dried at 333 K for 24 h. The final composites were ground into fine powders. They were then loaded into a Fe mold and pressed at 473 K under a pressure of 50 MPa for 5 min.

### 2.5 Characterization

X-ray diffraction (XRD; New D8 Advance, Bruker AXS) was used to characterize the crystalline structure of the synthesized materials. The analysis was performed at 40 mA and 40 kV at a scan rate of 1° s^−1^, with 2*θ* ranging from 5 to 70°, using Cu Kα radiation (*λ* = 0.154056 nm). The binding energy peaks of the synthesized materials were analyzed by X-ray photoelectron spectroscopy (XPS; VG-Microtech, ESCA2000). The morphologies and structural properties of the as-prepared products were analyzed by field-emission scanning electron microscopy (FE-SEM, SIGMA, Carl Zeiss). The elemental composition of the samples was analyzed by energy-dispersive X-ray spectroscopy (EDS, NORAN system 7, Thermo Scientific). Fourier transform infrared (FT-IR, Bio-rad FTS-1465) spectra of the composites were obtained using pressed, disk-shaped pellets of the samples mixed with potassium bromide (KBr), with an average of 32 scans in the 500–4000 cm^−1^ wavenumber range A homemade device containing a pair of voltmeters and thermocouples was used to measure the Seebeck coefficient. The value was determined from the linear relationship between the thermal electromotive force (Δ*V*) and the through-plane temperature difference (Δ*T*) of the composite films (*S* = Δ*V*/Δ*T*). The electrical conductivity of the samples was measured using the 4-point-probe method and the thickness of the sample was measured using a digital micrometer. The thermal conductivity of the samples was determined using the formula *κ* = *αρC*_p_, where *α* is the thermal diffusivity, *ρ* is the bulk density, and *C*_p_ is the specific heat of the materials. The thermal diffusivity of the samples was measured using a LFA 447 Nanoflash (NETZSCH) instrument. The specific heat of the samples was measured *via* differential scanning calorimetry (DSC; 131 evo, Setaram Instrumentation) in the temperature range of −20 °C to 120 °C at a heating rate of 10 K min^−1^ under N_2_ atmosphere. All measurements were performed in the same direction to prevent anisotropy of the samples.

## 3. Results and discussion

**Fig. 1:**
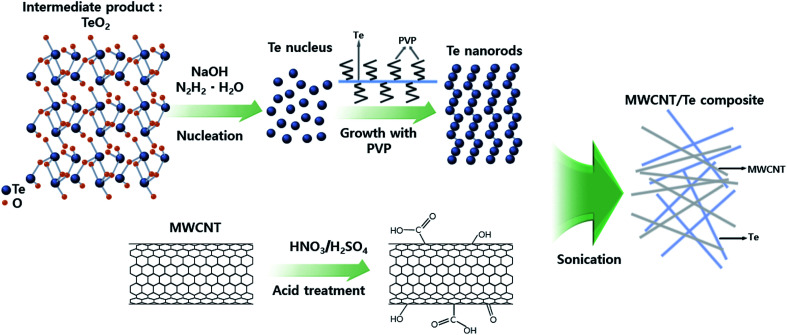
Schematic illustration of the fabrication of MWCNT/Te nanorod composite.

Fig. 1 shows the overall scheme of the synthesis of MWCNT/Te nanorod composite. TeO_2_, NaOH, and PVP were mixed with EG and heated until the temperature of the solution reached 393 K; later, N_2_H_4_·H_2_O was injected into the solution. PVP plays a key role as a surfactant in the synthesis of Te nanorod. Previous studies have reported that a linear polymer can react with inorganic ions to form chain-shaped intermediates.^32,33^ However, in this study, PVP might also serve as a directing template for the synthesis of nanorod. In other words, PVP induces 1D growth and controls the growth rate of the Te nanorods. At this stage, the nucleation of the Te^2−^ ions occurred and they were reduced to elemental Te to form solid crystal nuclei. As the reduction continued, the growth of Te ions to Te nanorods was accelerated.

**Fig. 2:**
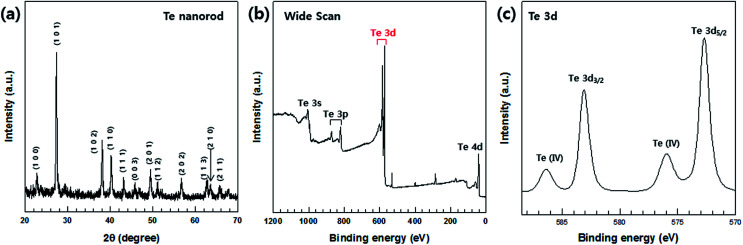
(a) XRD patterns and (b and c) XPS spectra of Te nanorods. (b) Wide XPS scan for Te. (c) Te 3d spectrum.

XRD analysis was conducted to determine the crystalline phases of the synthesized Te nanorods and the results are shown in Fig. 2(a). All the peaks shown in these patterns can be indexed to the hexagonal phase of Te (JCPDS, no. 13-1452) and no other peaks could be found, indicating that only elemental Te grains with high crystallinity were obtained.

The synthesized Te nanorods were further analyzed by XPS. The XPS data corresponding to the Te nanorods is shown in Fig. 2(b) and (c). A high-resolution spectrum of the Te 3d region (Fig. 2(c)) shows peaks at about 583.38 eV and 572.98 eV, which correspond to the Te 3d_3/2_ and Te 3d_5/2_ binding energies of Te, respectively. In addition, the two peaks at 586.88 eV and 576.48 eV (Fig. 2(c)) can be assigned as Te(iv) 3d binding energy, which indicates the oxidation of Te. It demonstrates that the outer surface of the Te nanorods is easily oxidized in air due to their 1D nanostructure. It can be concluded that the atoms at the surface have a lower coordination number and hence are more active than the inner atoms. Owing to this feature, the nanostructures could be more easily oxidized in air. Several previous studies reported that nanomaterials could be oxidized easily in air.^34^ Our XPS data is consistent with such results.^4^ This implies that the Te nanorods were successfully synthesized by the chosen fabrication process, which was further confirmed by FE-SEM and EDS analyses, the details of which are provided in the following sections.

**Fig. 3:**
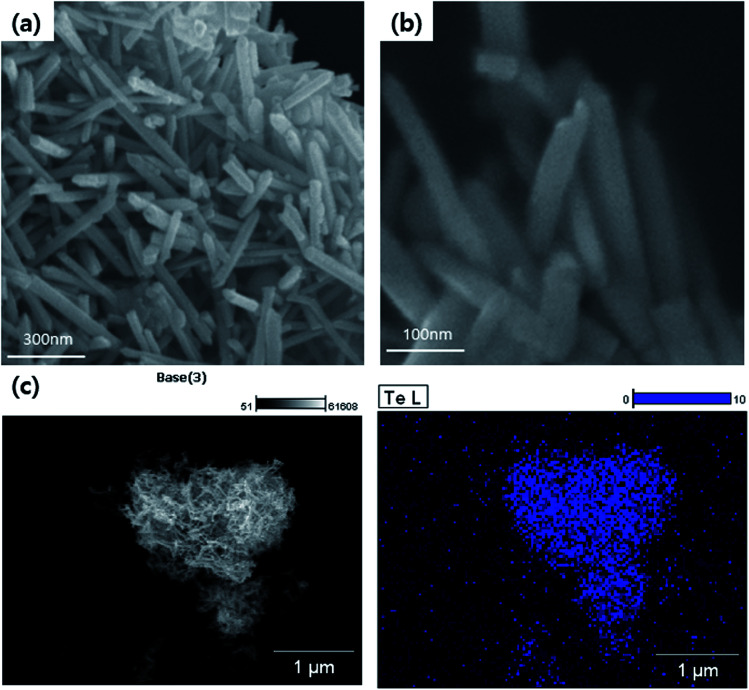
(a) Low and (b) high-magnification FE-SEM images of Te nanorods. (c) Particle surface morphology and atomic mapping of Te nanorods.

The fabricated 1D Te nanorod samples are clearly visible in the FE-SEM images shown in Fig. 3(a) and (b), which show the presence of a large number of randomly distributed wire-like structures. It can be observed that the obtained products are mostly cylindrically shaped rods of relatively uniform size. Each Te nanorod is ∼15 nm in length, and ∼600 nm in diameter. FE-SEM analysis thus confirms that Te nanorods were successfully prepared. This result was further verified by EDS atomic mapping for Te (Fig. 3(c)). The EDS spectrum collected from a specific region (Fig. 3(c)) indicated the presence of Te. From these results, we can infer that Te nanorods were successfully synthesized.

Prior to the synthesis of MWCNT/Te composites, the surfaces of the MWCNTs were treated by HNO_3_ and H_2_SO_4_. The treatment resulted in the formation of carboxylic groups on the surface of MWCNTs while keeping the MWCNT length intact. This reaction also leads to the elimination of impurities. Additionally, the carboxyl groups on the surface enhance the reactivity of MWCNTs, so this treatment make MWCNTs with Te nanorod easy.

**Fig. 4:**
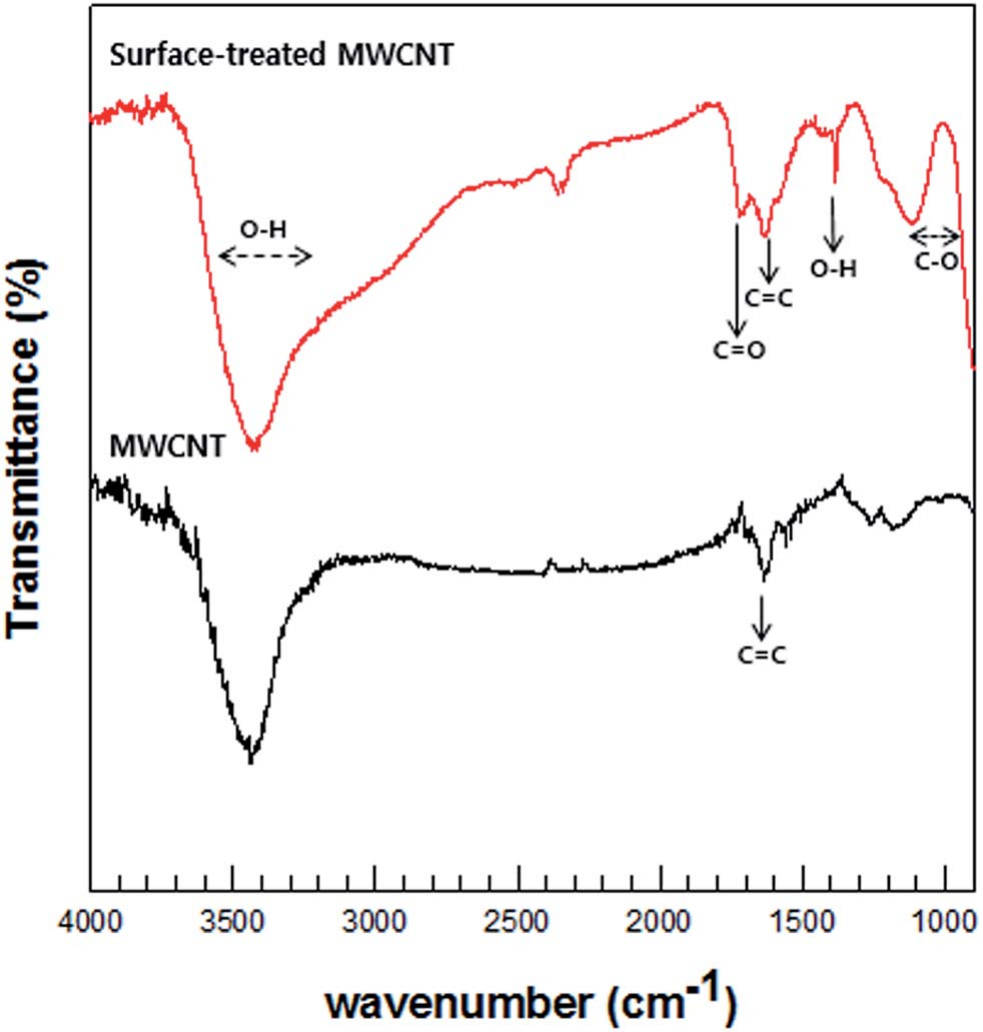
Typical FT-IR spectra of pristine MWCNT and surface-treated MWCNT.

FT-IR was performed to analyze the chemical bonding and types of functional groups grafted onto the MWCNTs. Fig. 4 illustrates the FT-IR spectra of the MWCNTs and acid-treated MWCNTs. Although the quality of the spectra is low because of the MWCNT texture, the data substantiate the formation of carboxyl group on the surfaces of the MWCNTs. The MWCNT spectrum denotes O–H stretching vibrations as a broad absorption band at 3400 cm^−1^ and as a relatively narrow peak at 1410 cm^−1^. The absorption peak at 1715 cm^−1^ corresponds to the typical stretching vibration of the C=O groups in the carboxyl units. The characteristic peak at about 1636 cm^−1^ can be assigned to the vibration of the aromatic C=C bonds. The broad peaks at 1180 cm^−1^ could be assigned to the C–O stretch of the alkoxy group.^35^

**Fig. 5:**
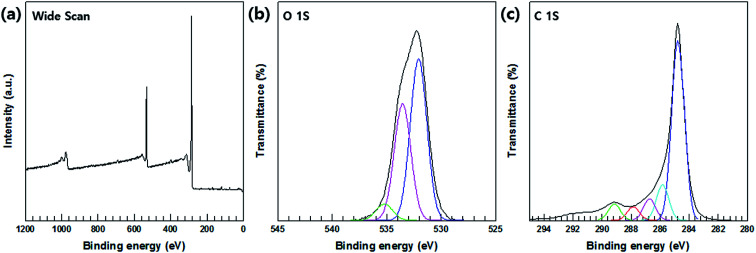
XPS spectra surface-treated MWCNTs. (a) Wide XPS scan (b) O 1s spectrum. (c) C 1s spectrum.

XPS analysis was relied upon to confirm the oxidation of the MWCNTs; the wide-survey and high-resolution spectra of the MWCNTs are shown in Fig. 5(a)–(c), respectively. The C 1s region of the XPS spectrum reveals the presence of C–C bonding in MWCNTs at a binding energy 284.6 eV, C–O bonding at 287.4 eV, C–H bonding at 285 eV, and C=O bonds of the carboxylated group at 288 eV; furthermore, at a binding energy of 289 eV, carbonyls consistent with carboxylated groups were formed.

**Fig. 6:**
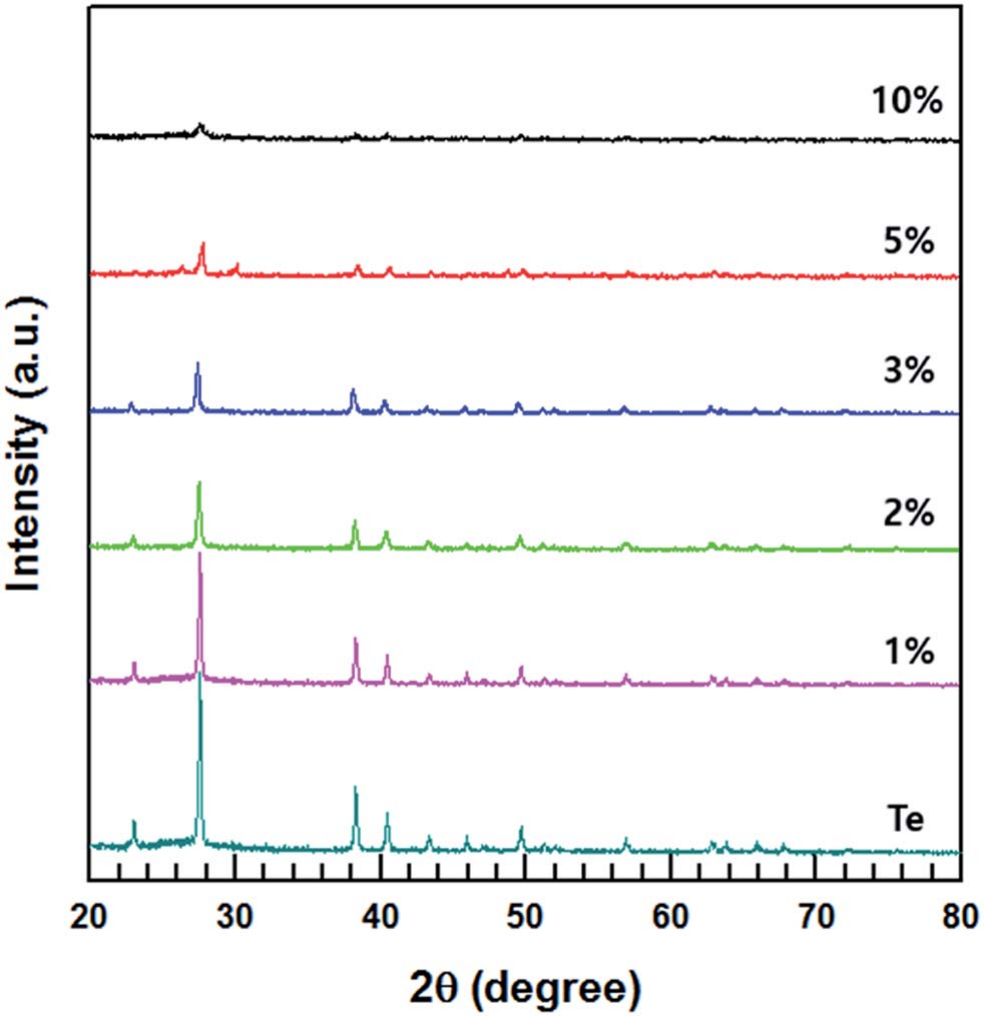
XRD patterns of MWCNT/Te nanorod composites with different MWCNT contents and Te nanorods.

The XRD patterns of the MWCNT/Te composites with various MWCNT contents (1, 2, 3, 5 and 10%) are shown in Fig. 6. The diffractograms of the composite are almost similar to that of the Te nanorods. However, with an increase in the MWCNT content, the intensities of the peaks corresponding to Te decrease in the XRD patterns of the composites.

**Fig. 7:**
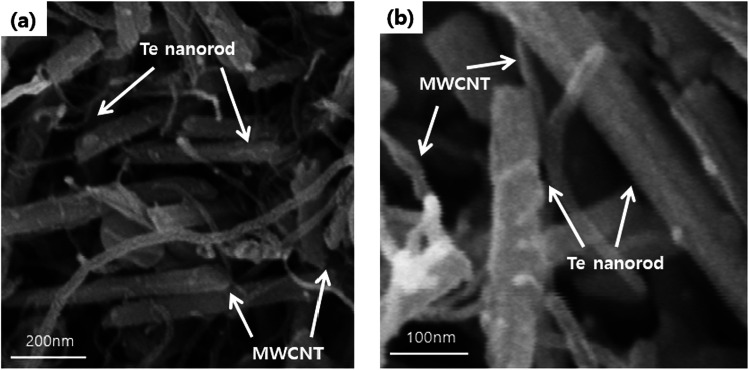
(a) Low and (b) high-magnification FE-SEM images of MWCNT/Te nanorod composites with 2 wt% MWCNT.

**Fig. 8:**
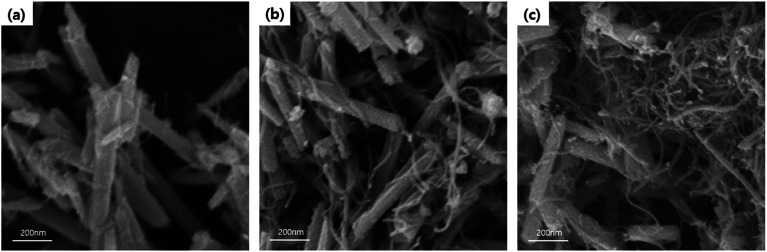
FE-SEM images MWCNT/Te nanorod composites with various MWCNT contents (a) 1 wt%, (b) 3 wt%, and (c) 5 wt%.

FE-SEM analysis was carried out to observe the microscopic morphology of the fabricated MWCNT/Te nanorod composites (Fig. 7 and 8). Fig. 7 shows the low and high magnification cross-sectional FE-SEM images of the MWCNT/Te nanorod composites 2 wt% of MWCNT contents. These images confirm that the MWCNTs were well dispersed in the Te nanorod matrix. However, after 3 wt%, MWCNT dispersion in Te matrice is not uniform, from 5 wt%, MWCNT aggregates themselves. These trend is confirmed at Fig. 8.

Prior to measuring the TE characteristics, using a hot press, the MWCNT/Te nanorod composites were fabricated into disks of 12.7 mm diameter; pure Te disks were also prepared.

**Fig. 9:**
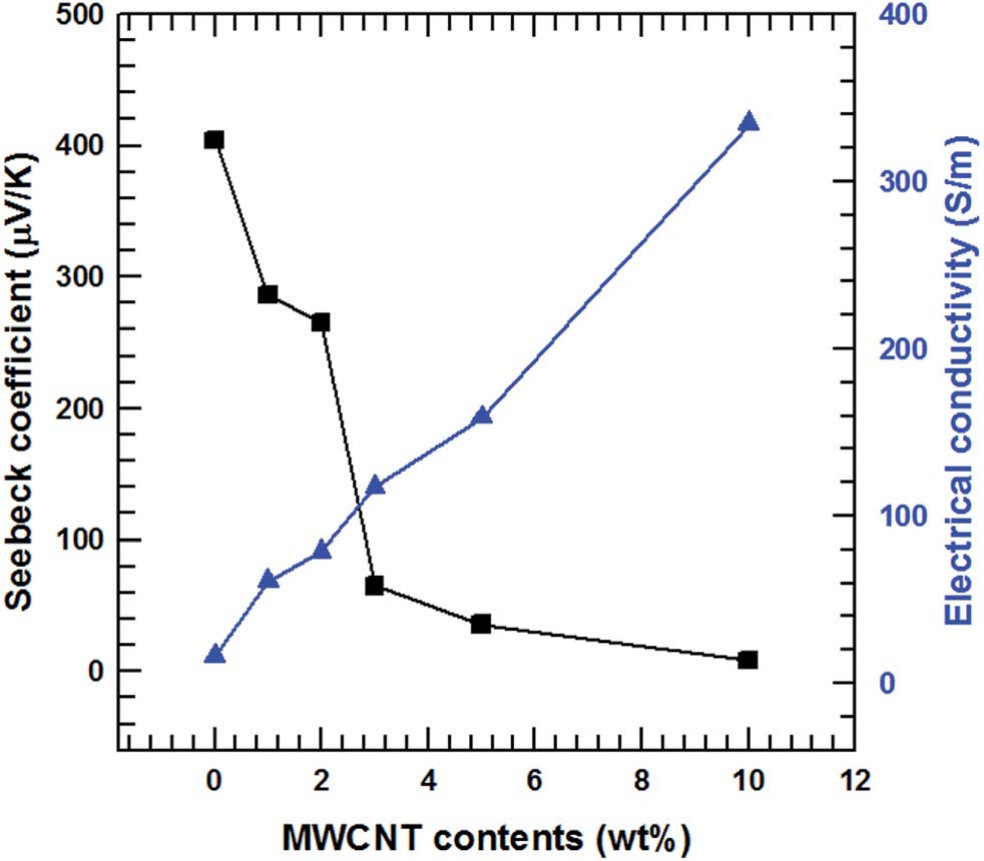
Seebeck coefficient and electrical conductivity of the MWCNT/Te nanorod composite with different MWCNT contents.

The electrical conductivities of the MWCNT/Te nanorod composite (1, 2, 3, 5 and 10%) at room temperature are shown in Fig. 8. The electrical conductivity of the pure Te sample was measured (Fig. 9) to be 16.3 S m^−1^. Pristine CNTs exhibit a very high electrical conductivity,^36^ which is much higher than that of the pure Te sample. As shown in Fig. 9, the electrical conductivity of the composites increases with increasing MWCNT content and at 10 wt%, the maximum value of 334.2 S m^−1^ was obtained. This increasing trend in electrical conductivity is attributed to the electrical conductivity of MWCNTs, which is relatively higher than that of the Te nanorods.

The Seebeck coefficients of the MWCNT/Te nanorod composites containing 1, 2, 3, 5 and 10% MWCNT at room temperature are also shown in Fig. 9. Unlike the electrical conductivity, the Seebeck coefficients experience a decrease with an increase in the MWCNT content. The Seebeck coefficient of the pristine Te nanorods is 404.5 μV K^−1^, which is similar to the value reported previously.^3,4^ The Seebeck coefficient of pure MWCNTs is ∼12.2 μV K^−1^, which is analyzed in previous studies;^36^ the Seebeck coefficient of MWCNTs is much lower than that of the Te nanorods. The decreasing trend observed in the Seebeck coefficients of the composite samples is due to the difference in the Seebeck coefficients of the two materials. In addition, the Seebeck coefficients of both MWCNTs and Te nanorod have positive values, which indicates that the two materials exhibit p-type electrical transport behavior.

### 3.1 Nanorod matrix to form a composite

**Fig. 10:**
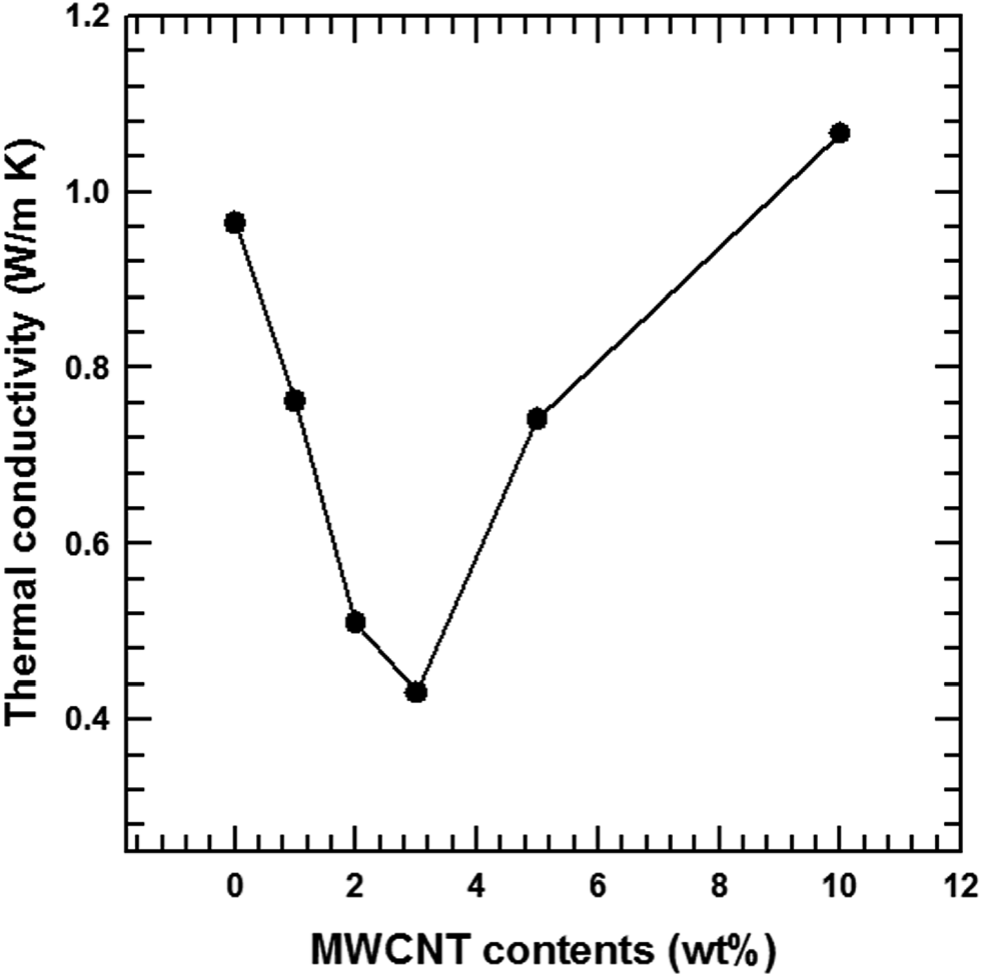
Thermal conductivity of the MWCNT/Te nanorod composites with different MWCNT contents.

Fig. 10 illustrates the dependence of the total thermal conductivity of the composites on the MWCNT content. The total thermal conductivity (*κ*) consists of a lattice thermal term (*κ*_l_) corresponding to phonons and an electronic term (*κ*_e_) corresponding to the charge carriers (*κ* = *κ*_l_ + *κ*_e_). *κ*_e_ is estimated from the Wiedemann–Franz law *κ*_e_ = *LσT* where *L* is the Lorentz number (*L* = 2.45 × 10^−8^ W Ω K^−2^).^37,38^ The total thermal conductivity is mainly dependent on the lattice term *κ*_l_ because of the relatively small contribution of the electronic term. Thus, the lattice scattering of phonons is the dominant factor that determines *κ* for the composite. The findings in this study show that the incorporation of MWCNT particles into the Te matrices generated MWCNT/Te heterointerfaces, providing effective phonon-scattering centers. Therefore, the *κ*_l_ values of the composites were lower than those of the pure Te samples. The *κ* value of the pristine Te sample decreased until the MWCNT reached 3 wt% due to the phonon scattering effect. However, above 3 wt% of MWCNTs, the *κ* values tended to increase because of the outstanding thermal conductivity of the MWCNTs.^39,40^ This value overwhelms the reduction in the *κ* value resulting from phonon scattering.

**Fig. 11:**
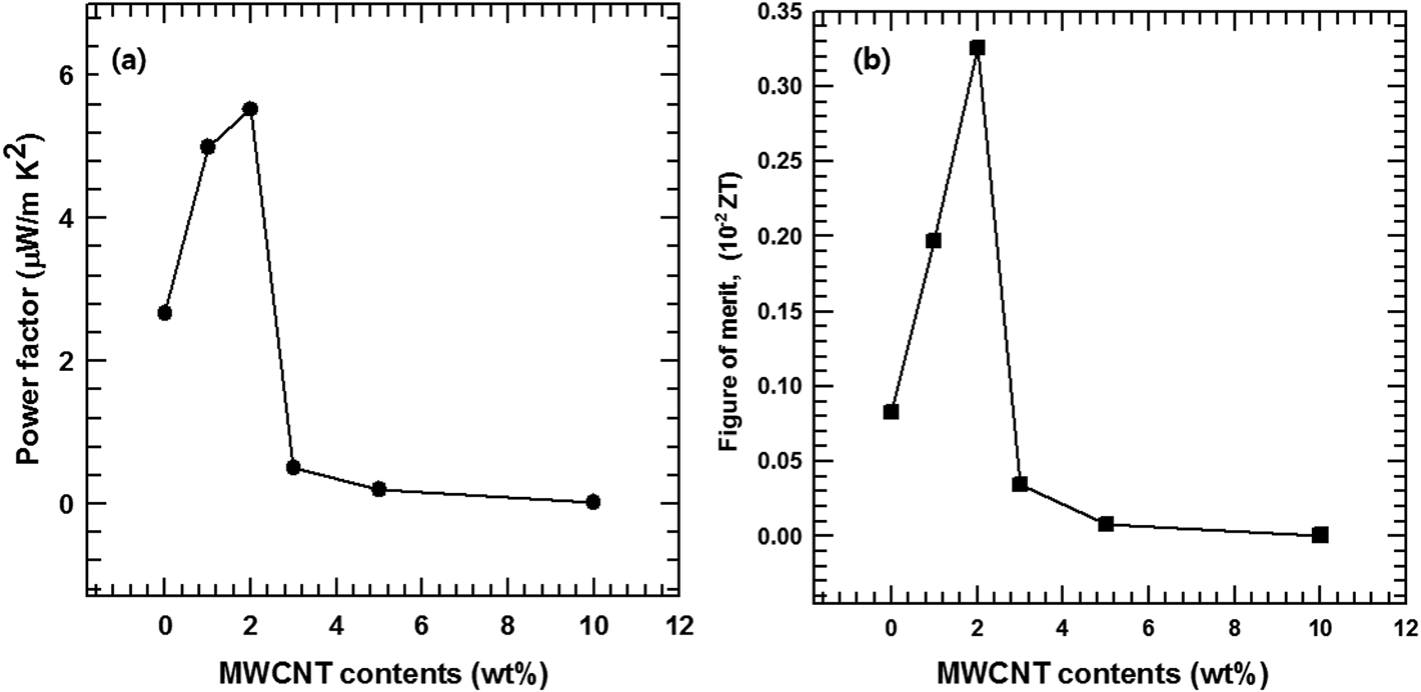
(a) Power factor and (b) figure of merit (*ZT*) of the MWCNT/Te nanorod composite with different MWCNT contents.

The thermoelectric performances of the MWCNT/Te nanorod composites were evaluated by analyzing the power factor as well as the thermoelectric figure of merit (*ZT* = *S*^2^*σT*/*κ*). The power factors of the MWCNT/Te nanorod composites with various MWCNT contents are shown in Fig. 11(a). The composite sample with 2% MWCNT shows the highest power factor (5.53 μW m K^−2^). This value is ∼2.07 times larger than the power factor of pure Te nanorods. The addition of small amounts of MWCNTs to the composite resulted in a high power factor because of the high electrical conductivity of the MWCNTs. However, in composite samples with MWCNT contents greater than 2%, the power factor decreased due to the low Seebeck coefficient of the MWCNT.

The thermoelectric figure of merit, *ZT*, was also evaluated and the results are shown in Fig. 11(b). Due to the improved power factor and low thermal conductivity of the MWCNT/Te nanorod composites, the *ZT* values of the composites are much higher than that of the pristine Te nanorods. At room temperature, the maximum *ZT* value of 4.5 × 10^−3^, was exhibited the composite with 2% MWCNT, which is ∼3.91 times larger than that of pure Te nanorods. These results indicate that the introduction of MWCNTs can synergistically enhance the thermoelectric properties of Te nanorods.

The MWCNT/Te nanorod composites studied in this investigation exhibited improved thermoelectric characteristics. The high electrical conductivity of the MWCNTs can enhance the thermoelectric properties of the composites. In addition, the thermal conductivities of the MWCNT/Te nanorods composites were found to be low, owing to the strong phonon scattering at the nanostructured interfaces between the nanorods and the MWCNTs. The results of this study suggest that the synthesis of synergetic networks consisting of Te nanorods and MWCNTs can enhance the thermoelectric properties of the MWCNT/Te composites.

## 4. Conclusion

MWCNT/Te nanorod composites with varying MWCNT contents were fabricated, and their thermoelectric properties were investigated. During the solution-phase synthesis of Te nanorods, PVP helps in the structuring of wire-like Te structure. It holds the each particles, so the particles grow with 1D nanostructure. The MWCNTs used in this study were treated with an acidic solution of H_2_SO_4_ and HNO_3_ to generate carboxylic groups on their surfaces. The MWCNTs were uniformly distributed throughout the Te nanorod matrix by ultrasonication and vacuum filtering, providing a well-dispersed solution. The nanostructures and morphologies of the samples were evaluated by FE-SEM, EDS, XRD, XPS and FT-IR analyses. We also investigated the effect of composite formation on the thermoelectric properties of the Te nanorods. The Seebeck coefficient of the composite decreased with increasing MWCNT content. However, the electrical conductivity of the composites increased with an increase in the MWCNT content. As a result, composites with 2% MWCNT exhibited the largest power factor of 5.52 μW m K^−2^ at room temperature. This value is ∼200% higher than that of pure Te nanorods. The reduction in the thermal conductivity of the composites is attributed to the phonon scattering effect. 1D nanostructures exhibit increased phonon scattering, as a result of which the lattice thermal conductivity is reduced. The dependence of thermal conductivity on the MWCNT content was also analyzed. Te highest figure of merit was obtained with the composite containing 2% of MWCNT. It is ∼3.91 times larger than that of pure Te nanorods.

## Conflicts of interest

There are no conflicts to declare.

## Supplementary Material
