# Correction: A p-type multi-wall carbon nanotube/Te nanorod composite with enhanced thermoelectric performance

**DOI:** 10.1039/d0ra90025g

**Published:** 2020-03-20

**Authors:** Dabin Park, Hyun Ju, Taeseob Oh, Jooheon Kim

**Affiliations:** School of Chemical Engineering & Materials Science, Chung-Ang University Seoul 06974 Republic of Korea jooheonkim@cau.ac.kr

## Abstract

Correction for ‘A p-type multi-wall carbon nanotube/Te nanorod composite with enhanced thermoelectric performance’ by Dabin Park *et al.*, *RSC Adv.*, 2018, **8**, 8739–8746.

The authors regret that an incorrect version of Fig. 8 was included in the original article. The correct version of Fig. 8 is presented below.

**Fig. 1:**
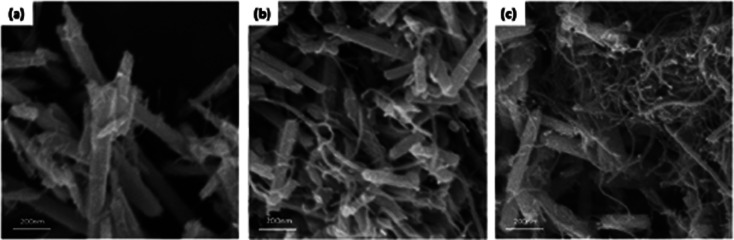
FE-SEM images of MWCNT/Te nanorod composites with various MWCNT contents (a) 1 wt%, (b) 3 wt%, and (c) 5 wt%.

The Royal Society of Chemistry apologises for these errors and any consequent inconvenience to authors and readers.

## Supplementary Material

